# Uncertain magnitude of benefit of early second-generation androgen receptor antagonist treatment in advanced prostate cancer

**DOI:** 10.3389/or.2026.1750202

**Published:** 2026-05-12

**Authors:** Emma K. van de Weerdt, Ashley J. Duits, Michael J. Samson, John-John B. Schnog

**Affiliations:** 1 Department of Hematology-Medical Oncology, Curaçao Medical Center, Willemstad, Curaçao; 2 Curaçao Biomedical and Health Research Institute, Willemstad, Curaçao; 3 Department of Medical Education, Curaçao Medical Center, Willemstad, Curaçao; 4 Institute for Medical Education, University Medical Center Groningen, Groningen, Netherlands; 5 Red Cross Blood Bank Foundation, Willemstad, Curaçao; 6 Department of Radiation Oncology, Curaçao Medical Center, Willemstad, Curaçao

**Keywords:** androgen receptor signaling inhibitor, bias, chemotherapy, guideline, health technology assessment, prostate cancer, randomized controlled trials

## Abstract

Guidelines currently recommend the early use of second-generation androgen receptor signaling inhibitors (ARSIs) in both castrate-resistant and -sensitive metastatic prostate cancer (with or without chemotherapy) and also in biochemical non-metastatic castrate-sensitive relapse. We highlight design issues in the randomized controlled trials (RCTs) that bias the results of early ARSI use, suggesting that the magnitude of benefit might be overestimated and, therefore, uncertain. Issues include sub-optimal post-protocol care, inadequate control arms, and the inclusion of highly selected patients, which are increasingly characteristic of contemporary cancer RCTs. The uncertainty regarding the true magnitude of benefit of early ARSI use complicates health technology assessment (HTA). Recognition of such trial design issues with their resultant magnitude of benefit uncertainty is important not only for individual patient care but also for cancer policy decision-making in general.

## Introduction

It is increasingly recognized that design issues in contemporary randomized controlled trials (RCTs) in cancer care, such as sub-optimal post-protocol care and inadequate control arms, may bias trial results in favor of the experimental arm, while the universal use of putative surrogate endpoints and inclusion of highly selected patients make it difficult to estimate the true real-world benefit of the intervention under investigation. The importance of the efficacy–effectiveness gap, which refers to the inferior outcome of cancer interventions in the real world (effectiveness) as opposed to the outcome achieved within the constraint of an RCT (efficacy), should be taken into account for both individual patient care and decision-making on cancer policy (1). Without careful consideration of design and interpretation issues that bias results to the experimental arm in RCTs, the true benefit of cancer interventions can be overestimated. It is of increasing importance that clinicians recognize this in order to properly guide patients’ decisions about treatment as well as contribute to broader cancer policy decision-making (2, 3). Globally, cancer policy decisions are dependent on many different factors, such as available resources and financial constraints. Importantly, what is considered a relevant improvement in outcomes will vary between cultures and countries (4). As an instructive example, we detail issues that have occurred across more than a decade of prostate cancer RCTs on androgen receptor signaling inhibitors (ARSIs) that have biased results of the experimental arms.

**FIGURE 1:**
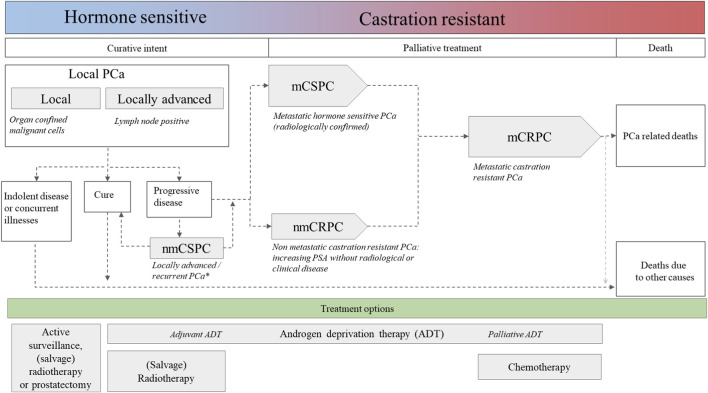
Overview of prostate cancer progression and treatment options across disease stages. The flowchart illustrates prostate cancer (PCa) progression from localized hormone-sensitive disease to metastatic castration-resistant prostate cancer (CRPC). Treatment strategies are shown across disease stages, including active surveillance, prostatectomy, radiotherapy, androgen deprivation therapy (ADT), androgen receptor signaling inhibitors (ARSIs), and taxane-based chemotherapy. Radionucleotide treatment options are not depicted.

Prostate cancer is the most frequently diagnosed malignancy among men (5). The majority of patients present with localized prostate cancer. Based on risk stratification models, these patients can be treated with curative intent (surgery or radiotherapy) or managed with a conservative strategy of active surveillance. Patients with metastatic prostate cancer are generally incurable and are initially treated with androgen deprivation therapy (ADT). *De novo* high-volume metastatic prostate cancer patients have been demonstrated to benefit from an upfront combination of ADT with six cycles of taxane-based chemotherapy (docetaxel) (6). Despite initial responsiveness to ADT, progression to castration-resistant prostate cancer (CRPC) is inevitable. Until the advent of ARSIs such as abiraterone and enzalutamide, patients with metastatic CRPC (mCPRC) were treated with taxane-based chemotherapy docetaxel (FDA approved in 2004 (7, 8)), with cabazitaxel becoming available later as a second-line chemotherapy (FDA approved in 2010 (9)) (see Figure 1)

Since then, ARSIs have come to play a prominent role in the treatment of prostate cancer by moving their use to earlier stages of disease progression. In this narrative review, we argue that, despite more than a decade of important RCTs on the earlier use of ARSIs in prostate cancer patients, the true magnitude of benefit of early ARSI use is likely overestimated and thus remains undefined. “Early ARSI use” is defined as any use prior to docetaxel in metastatic settings, including both metastatic castration-resistant prostate cancer (mCRPC), metastatic castration-sensitive prostate cancer (mCSPC), and localized PCa.

### Evolving role of ARSIs in the treatment of prostate cancer

RCTs first showed that ARSIs improve overall survival (OS) in patients with mCPRC following docetaxel compared to placebo (COU-AA-301 (10–12) and AFFIRM (13)). In the years following, RCTs have investigated ARSIs before chemotherapy in patients with mCRPC (COU-AA-302 (14, 15) and PREVAIL (16, 17)). Several trials were performed in patients with newly diagnosed high-risk hormone-sensitive metastatic prostate cancer (mCSPC, referred to in some trials as “metastatic hormone-sensitive prostate carcinoma” —mHSPC) (STAMPEDE (18, 19), LATITUDE (20, 21), and ARCHES (22, 23)). In mCSPC patients, abiraterone was assessed with ADT and (a later amendment) docetaxel ( ± radiotherapy to the primary tumor) in PEACE-1 (24) and as first line therapy in ENZAMET (25–27). Next-generation ARSIs apalutamide (with ADT) and darolutamide (with docetaxel and ADT as a backbone) have also been studied in mCSPC (ARASENS (28), ARANOTE (29), and TITAN (30)). Recent trials have been performed in patients with biochemical recurrent prostate carcinoma while receiving ADT (PROSPER (31, 32), SPARTAN (33), and ARAMIS (34)) in castrate sensitive biochemical recurrence after treatment (EMBARK (35)).

When treatments are moved to earlier lines, RCTs should clearly demonstrate an improvement in OS, quality of life (QOL), or both (36). This is especially important as per cancer biology, treatment duration is often longer when cancer drugs are given earlier in the disease course, leading to prolonged treatment exposure with related side effects as well as increased healthcare expenditure. In general, when cancer drugs which are efficacious in late-stage cancer are tested in earlier disease stages, (putative) surrogate endpoints are used as primary outcomes that do not always predict clinical benefit (2). The control arms in RCTs should reflect the standard of care at the time of trial initiation (2). Furthermore, when testing a proven life-prolonging cancer drug in an earlier line of treatment (for example, moving treatment up from second- to first-line palliative), crossover to the drug in question should be mandatory after patients in the control arm progress (2) and should occur within the constraints of the RCT, employing the same rigorous predefined outcome assessments and toxicity monitoring and applying the same stopping rules used at initial randomization. Only then can a RCT determine whether earlier initiation of a drug is better as opposed to its use in a later stage of disease. A recent example of a well-designed RCT was the SONIA trial, in which first- versus second-line cyclin-dependent kinase 4 and 6 inhibitors did not confer benefit, with second-line use shorter and thus more cost-effective (37). Ideally, patients included in the trial should reflect, as closely as possible, real-world patients to provide results applicable to everyday practice (2). It is, however, increasingly recognized that the modern landscape of cancer RCTs is characterized by shortcomings that often provide suboptimal data on which drug regulatory agencies approve new cancer drugs and drug indications (2). With a low bar for regulatory drug approvals, many cancer drugs with limited or unproven benefit are reaching the market (4). This challenges global health technology assessment (HTA) organizations in their effort to adequately prioritize reimbursement for treatments with significant value over those demonstrating limited to no benefit following regulatory agency approvals.

With prostate cancer incidence projected to steeply increase worldwide, the judicious use of available drugs and the optimal use of healthcare resources are imperative (38). Regulatory agencies have mostly approved early ARSI use and professional guideline incorporation of ARSIs in pre-chemotherapy CRPC and hormone-sensitive advanced prostate cancer (Table 1). For this narrative review, we identified 16 RCTs (Table 2) that, at the time of writing, formed the basis for regulatory approval of the studied ARSI indications by the Food and Drug Administration (FDA), the European Medicines Agency (EMA), or both. We performed structured searches in PubMed to identify all RCTs that led to regulatory approval of an ARSI in earlier treatment lines. Landmark registration trials and their primary publications were cross-referenced to ensure completeness. We systematically searched for long term follow up/final analysis of the respective trials to include final OS survival data. The aim was not to conduct a formal systematic review or meta-analysis but to provide a critical appraisal of the design and interpretability of these RCTs.

**TABLE 1:** Lists of approvals (FDA and EMA) and whether the indication is included in the latest NCCN and ESMO guidelines.

Indication (abiraterone)					
​	​	Approval		Guideline uptake	
Indication	Trial	FDA (96)	EMA (97)	NCCN (98)	ESMO (99)
mCSPC	STAMPEDE, LATITUDE PEACE-1	2018	2018	Yes	Yes
mCRPC prior to chemotherapy	COU-AA-302	2012	2013	Yes	Yes
mCRPC post chemotherapy	COU-AA-301	2011	2011	Yes	Yes

Indications enzalutamide					
​	​	Approval		Guideline uptake	
Indication	Trial	FDA (39)	EMA (40)	NCCN (41)	ESMO (42)
PCa biochemical recurrence PSADT <10 months, nmCSPC	EMBARK	2023	2024	Yes	Yes
nmCRPC	PROSPER	2018	2018	Yes	Yes
mCSPC	ARCHES ENZAMET	2019	2021	Yes	Yes
mCRPC prior to chemotherapy	PREVAIL	2014	2014	Yes	Yes
mCRPC post chemotherapy	AFFIRM	2012	2013	Yes	Yes

Indications apalutamide					
​	​	Approval		Guideline uptake	
Indication	Trial	FDA (39)	EMA (40)	NCCN (41)	ESMO (42)
nmCRPC	SPARTAN	2018	2019	Yes	Yes
mCSPC	TITAN	2019	2019	Yes	Yes

Indications darolutamide					
​	​	Approval		Guideline uptake	
Indication	Trial	FDA (39)	EMA (40)	NCCN (41)	ESMO (42)
nmCRPC	ARAMIS	2019	2020	Yes	Yes
mCSPC with docetaxel	ARASENS	2022	2023	Yes	No
mCSPS	ARANOTE	2025	2025	Yes	No

PCa, prostate cancer; PSADT, prostate-specific antigen doubling time; PSA, prostate-specific antigen; CRPC, castration-resistant prostate cancer; nmCRPC, non-metastatic castration-resistant prostate cancer; mCRPC, metastatic castration-resistant prostate cancer; mCSPC, metastatic castration-sensitive prostate cancer; FDA, US Food and Drug Administration; EMA, European Medicines Agency; NCCN, National Comprehensive Cancer Network, ESMO, European Society for Medical Oncology.

**TABLE 2:** Primary outcomes of trials.

Trial	Experimental arm	Control arm	Blinding	Disease setting	ECOG 0 + 1^@^	Primary outcome	Effect size (experimental vs. control) primary outcome
COU-AA-301 (10–12)	Abiraterone + prednisone + ADT	Placebo + Prednisone + ADT	Blinded	mCRPC	89.4%	OS	OS: 15.8 vs. 11.2 months; rPFS: 5.6 vs. 3.6 months
COU-AA-302 (14)	Abiraterone + prednisone + ADT	Placebo + Prednisone + ADT	Blinded	mCRPC	100%	rPFS and OS	rPFS: 16.5 vs. 8.3 months. OS not reached vs. 27.2 months; did not cross the efficacy boundary
STAMPEDE (18, 19)	Abiraterone + prednisone + ADT	ADT	Open label	Newly diagnosed mCSPC, relapsing PCa starting ADT, or high-risk locally advanced^$^	0: 78% 1–2: 22%^$^	OS	OS: 79 months vs. 46 months. The OS data from the STAMPEDE trial patients with mCSPC
LATITUDE (20, 21)	Abiraterone + prednisone + ADT	ADT	Blinded	mCSPC	96.7%	OS and rPFS	OS: 53.3 vs. 36.5 months; rPFS 33 vs. 14.8 months
PEACE-1 (24)	Abiraterone + prednisone + ADT + docetaxel*	ADT + docetaxel*	Open label	mCSPC	0: 70.1% 1–2: 29.9%^$^	OS and rPFS	OS: 5.72 vs. 4.72 years; rPFS 4.46 vs. 2.22 years
AFFIRM (13)	Enzalutamide + ADT	Placebo + ADT	Blinded	mCRPC	91.5%	OS	OS: 18.4 vs. 13.6 months
PREVAIL (16, 17)	Enzalutamide + ADT	Placebo + ADT	Blinded	mCRPC	100%	OS and rPFS	OS: 36 vs. 31 months
ARCHES (22, 23)	Enzalutamide + ADT	Placebo + ADT	Blinded	mCSPC	99.9%	rPFS	OS not reached vs. 47.7 months. At data cut-off, radiologic progression or death occurred in 15.9% of the enzalutamide group vs. 34.9% in the placebo group
PROSPER (32)	Enzalutamide + ADT	Placebo + ADT	Blinded	nmCRPC	100%	MFS, OS	MFS: 36.6 vs. 14.7 months. Final analysis. mOS: 67.0 vs. 56.3 months
ENZAMET (26, 27)	Enzalutamide + ADT	Bicalutamide, nilutamide, or flutamide + ADT	Open label	mCSPC	99.8%	OS	OS not reached. At data cutoff at 5 years, 37% of patients in the enzalutamide group died vs. 48% of patients in the control group
EMBARK (35)	Enzalutamide + ADT or enzalutamide monotherapy**	Placebo + ADT	Open label	PCa with biochemical recurrence, nmCSPC	99.9%	MFS	At 5 years, MFS was 87% in the combination group vs. 71% in the leuprolide-alone group and 80% in the enzalutamide monotherapy group
SPARTAN (33)	Apalutamide + ADT	Placebo + ADT	Blinded	nmCRPC	99.9%	MFS	Median MFS: 40.5 vs. 16.2 months
TITAN (30, 100)	Apalutamide + ADT	Placebo + ADT	Blinded	mCSPC	100%	rPFS, OS	rPFS at 24 months: 68.2% vs. 47.5% Median OS not reached vs. 52.2 months
ARAMIS (64)	Darolutamide + ADT	Placebo + ADT	Blinded	nmCRPC	100%	MFS	MFS: 40.4 vs. 18.4 months
ARASENS (28)	Darolutamide + docetaxel + ADT	Placebo + docetaxel + ADT	Blinded	mCSPC	99.8%	OS	OS at 4 years: 62.7% vs. 50.4%
ARANOTE (29)	Darolutamide + ADT	Placebo + ADT	Blinded	mCSPC	97%	rPFS	rPFS not reached vs. 25 months rPFS 70.3% vs. 52.1% at 24 months

@ denotes the percentage of patients included with ECOG (Eastern Cooperative Oncology Group) performance status 0 + 1.

^$^ Patients with ECOG 1–2 were reported together.

^¶^ OS data from STAMPEDE trial patients with mCSPC.

* Participants randomized (1:1:1:1) to standard care (ADT ± docetaxel), standard care plus radiotherapy, standard care plus abiraterone (with prednisone), or standard care plus both.

Primary outcomes of trials. ** In EMBARK, patients were randomized (1:1:1) to enzalutamide + leuprolide, placebo + leuprolide, or enzalutamide monotherapy.

ADT, androgen deprivation therapy; PCa, prostate carcinoma; CRPC, castration-resistant prostate carcinoma; mCRPC, metastasized castration-resistant prostate carcinoma; nmCRPC, non-metastasized castration-resistant prostate carcinoma (e.g., PSA increase without signs of radiologically metastasized disease; OS, overall survival; rPFS, radiological progression free survival; MFS, metastasis free survival.

### Suboptimal post-protocol care in control arm patients

The AFFIRM and COU-AA-301 trials demonstrated the OS benefit of ARSI use in mCPRC patients after progression on taxane-based chemotherapy. In subsequent trials conducted to investigate administering an ARSI in an earlier course of the disease (e.g., mCPRC prior to taxane chemotherapy, mCSPC, or even localized disease), patients in the control arm should all have crossed over to the studied ARSI at progression. Withholding cross-over to the experimental drug when its efficacy has been proven in the following treatment line is recognized as a frequent problem in oncology RCTs that confounds the interpretation of results. A recent analysis of published oncology RCTs in high impact journals (2018–2020) and FDA approvals in the same period showed that post-progression data are reported in less than half of RCTs and approvals. When reported, post-progression treatment was sub-standard in more than half of publications and in 75.7% of FDA approvals (43).

In trials studying abiraterone use before or concomitant with chemotherapy, between 42% (PEACE-1) and 78% (LATITUDE) of control-arm patients receiving post-protocol care never received abiraterone at the time of final publication (Supplementary Material) (20, 21, 24). In studies on earlier enzalutamide use pre-chemotherapy, 50% (ENZAMET) to 72% (ARCHES) of patients in the SOC of the trials never received enzalutamide at final publication (Supplementary Material) (22, 23, 25–27, 31). In the darolutamide RCTs, 5%–65% of patients never received an ARSI after disease progression at time of publication (28, 29, 34). For apalutamide, the range of patients to not receive an ARSI after disease progression was between 71% and 14% (30, 33). Even though chemotherapy post-progression is not the focus of our analysis, not only did almost one in five patients on the placebo arm in PEACE-1 not receive an ARSI post progression, but cabazitaxel exposure was also 17% lower in the control arm (24). Such factors will lead to an overestimated OS benefit. PREVAIL, PEACE-1, ENZAMET, TITAN, ARASENS, ARANOTE, and SPARTAN presented or allowed the calculation of the exact percentages of patients never to have received any ARSI in the control arm reported at time of publication—17%, 19%, 24%, 71,% 45%, 65%, and 14%, respectively (16, 17, 24, 26–30, 34).

**FIGURE 2:**
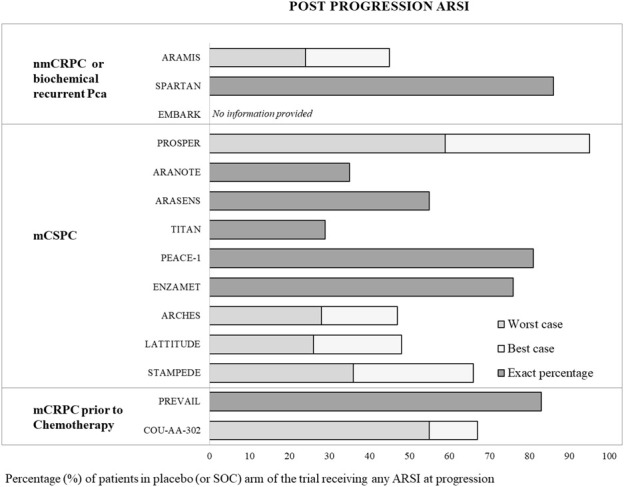
Post-protocol exposure to androgen receptor signaling inhibitors across prostate cancer trials. Bars show the percentage of patients in the placebo or standard-of-care arm who received an androgen receptor signaling inhibitor (ARSI) after disease progression. Trials are grouped by disease setting: non-metastatic castration-resistant prostate cancer (nmCRPC) or biochemically recurrent prostate cancer, metastatic castration-sensitive prostate cancer (mCSPC), and metastatic castration-resistant prostate cancer (mCRPC) prior to chemotherapy. Dark gray bars indicate exact reported percentages. When post-protocol ARSI exposure was incompletely reported, light gray bars represent the worst-case scenario, assuming different ARSIs were given to the same patients, whereas white bars represent the best-case scenario, assuming different ARSIs were given to different patients. No post-trial ARSI exposure data were available for EMBARK.

In the PEACE-1 trial, 81% of patients in the placebo-arm received an ARSI at progression, but the total of abiraterone and enzalutamide exceeded 100%, indicating that individual patients received both ARSIs post-progression. Treatment with enzalutamide after abiraterone, or *vice versa*, will not lead to substantial benefit and is not a recommended treatment with a generally poor outcome (44–47). With the best-case scenario assumption that abiraterone and enzalutamide, when both administered post-protocol to the control arm, were not administered to the same individuals (highly unlikely), the range of patients never to receive any ARSI post-protocol was 5%–53% (Supplementary Material). However, if the ARSIs were administered to the same patient (worst-case scenario), 17%–74% of patients never received an ARSI at progression (Figure 2). With such rates of control arm patients not receiving the life-prolonging standard of care after progression, it is likely that the reported OS benefit at time of publication is overestimated. This lack of specified crossover to the standard of care and life prolonging treatments in prostate cancer RCTs has also been addressed by others (48, 49).

### Control arm quality

The control arm of a trial should reflect the standard of care at the time of trial initiation. Empirical analysis has shown that in 2013–2018, approximately 17% of FDA approvals were based on RCTs in which the control arm was considered suboptimal (50). The use of a suboptimal comparator favors the experimental arm if the control treatment does not represent the best available therapy at the time of trial conduct.

In both RCTs that evaluated ARSIs in patients progressing after chemotherapy, placebo was used as the comparator. Although acceptable, this can be debated as several active, albeit modestly effective, treatment options were part of clinical practice at that time (51–53). Given that in the AFFIRM trial, at least 61% of patients in the placebo arm received post-progression therapy, a physician’s choice of comparator might have better reflected real-world practice than placebo alone. Post-trial treatment data were not available for the COU-AA-301 trial, but it is very likely that post-progression, as in the AFFIRM trial, many patients received active treatments. This indicates that the OS benefit attained in both COU-AA-301 and AFFIRM is likely overestimated, as many patients were on placebo when they could have received active therapy, albeit with limited effectiveness.

For both abiraterone and enzalutamide, the control arm of placebo was inappropriate when moving treatments to before chemotherapy in mCRPC, (15, 16). When questioning whether ARSI use prior to chemotherapy is superior than after chemotherapy in symptomatic patients, the control arm should have been the standard of care, which was docetaxel at the time (12, 16). These studies compared an active treatment to placebo in symptomatic patients with mCRPC. In the COU-AA-302 trial, 36% of patients in the placebo arm reported mild to moderate pain (patients with visceral metastases were excluded), and in the PREVAIL trial 33% of patients suffered from mild to moderate pain (patients with visceral metastases were included) surrogate rPFS in both studies is both obvious and non-informative (see below), with OS data yielding unreliable results based on the suboptimal post-protocol care detailed above. In PROSPER, which studied enzalutamide in non-metastatic castration-resistant prostate cancer (nmCRPC), the active treatment bicalutamide was stopped prior to randomization, which did not conform with contemporary guidelines (32, 50). Again, the observed improvement concerned a non-validated surrogate endpoint (rPFS) and was demonstrated in comparison with placebo, administered after the discontinuation of prior active therapy. The ADT control arm in TITAN was not appropriate, as the trial started accruing high-risk, mostly high-volume, and visceral metastatic patients long after superiority had been demonstrated for combining ADT with an ARSI, docetaxel, or both (54). In conclusion, symptomatic patients in the control arms of several of these RCTs did not receive optimal care, thus introducing bias favoring ARSIs in earlier treatment lines.

### Surrogate endpoints

Any medical intervention should improve survival, quality of life, or both. Hence, RCTs should use OS, QOL, or both as primary endpoints. Over recent decades, with the increasing rate of oncology RCTs sponsored by pharmaceutical companies, the use of (putative) surrogate endpoints such as “progression-free survival” (PFS) as primary endpoints has become ubiquitous (55, 56). It is often argued that use of surrogate endpoints in clinical trials would contribute to the faster availability of new treatments. However, an empirical analysis has demonstrated that the time gained by use of surrogates is relatively limited (57). For a putative surrogate endpoint to be considered a valid trial-level predictor of OS, QOL, or both, the German Institute for Quality and Efficiency in Health Care framework considers a correlation coefficient with the lower limit of the 95% confidence interval for R ≥ 0.85 (r^2^ ≥ 0.72) to be highly reliable, whereas an upper limit of the 95% confidence interval for R ≤ 0.70 (r^2^ ≤ 0.49) is considered invalid; values between these thresholds are deemed medium or uncertain (58). Furthermore, trial level putative surrogate endpoints should be validated prior to use per treatment line as well as per drug class. In general, surrogate endpoints are poor predictors of OS, QOL, or both (59–61). In a review on oncologic drug approvals based on surrogate endpoints, only 14% demonstrated improvement in OS in post-marketing follow-up (62).

In advanced prostate cancer, a recent meta-analysis of 143 randomized trials demonstrated the lack of validated for OS, QOL, or both (60). Only metastasis-free survival has been shown to predict OS in localized prostate cancer (63). Of the 16 RCTs addressed, 11 had OS as the primary or co-primary endpoint. Five trials had a surrogate endpoint as primary endpoint, with three studying metastasis-free survival (MFS) as the primary endpoint (33, 35, 64) and two trials reporting radiological PFS (rPFS) as the primary endpoint (Table 2). A surrogate endpoint is not needed when studying OS. The results pertaining to non-validated surrogate endpoints in these trials should not be used as proxies for the efficacy of moving the studied ARSIs to earlier lines of treatment.

### Selected patients

The effect of ARSI treatment in RCTs (efficacy) is based on highly selected patients who are not representative of daily clinical practice. Generally, patients participating in RCTs are younger, fitter, and have less comorbidity and fewer potential drug–drug interactions than everyday practice patients, as has also been demonstrated for prostate cancer trials (65–70).

The Eastern Cooperative Oncology Group (ECOG) performance status is a predictor of OS among patients with metastatic prostate cancer (71). In 8 of 16 RCTs, patients with an ECOG performance score > 1 were excluded (14, 16, 22, 32). In all trials, 70.1%–100% of patients had an ECOG score of 0–1. In two RCTs allowing ECOG 2 patients, the percentage of patients with ECOG 2 was not specified (29.9% of patients in PEACE-1 had ECOG 1–2, with ECOG 2 only allowed when due to bone pain; 22% of patients in STAMPEDE had 1–2) (19, 24). In trials reporting the percentage of ECOG two patients, only 0.1%–10.6% of patients were ECOG 2. This contributes to the efficacy–effectiveness gap (1).

### Treatment duration and costs

As argued above, the most reliably determined magnitude of benefit of ARSI use in prostate cancer is in patients with mCRPC after docetaxel. With earlier ARSI administration, time for treatment increases from 7.4 months in mCRPC after chemotherapy to as long as 58 months in mCSPC (see Supplementary Table) (10, 26). This impacts risk of toxicity as well as healthcare spending both at the societal and individual levels due to potential out-of-pocket costs (72).

Prolonged ARSI administration carries potential risks, including toxicities such as epileptic seizures (seen in 0.5% of trial patients with enzalutamide) and a higher rate of serious adverse cardiovascular events clearly related to the duration of ARSI exposure (73, 74). Fatigue emerges as a prominent side effect of enzalutamide treatment. A prospective observational study noted a slightly higher fatigue incidence than trial data (AFFIRM: 34% vs. 29%, PREVAIL 36% vs. 26% for enzalutamide vs. standard care respectively) (75). Real-world fatigue rates were substantially higher (54.7%) and correlated with treatment duration (75).

Financial distress over expensive medication likely negatively affects QOL, the effect of which is not captured in contemporary QOL analysis in oncology RCTs, as drugs provided in the trial are funded by the sponsoring pharmaceutical company (76). In the United States (US), enzalutamide as first-line treatment in patients with mCSPC was calculated to have a cost–effectiveness ratio of $430,934 per quality-adjusted life year (QALY) and $255,444 in China (77). At a willingness-to-pay threshold of $100,000 per QALY in the US, enzalutamide as a first line treatment of mCSPC is not cost effective (77). Even though abiraterone is now off patent, costs can still be an issue in many countries, and prolonged use also carries the burden and costs of more intensive monitoring and those related to adverse effects (78–80). Furthermore, with the advent of newer second generation ARSIs, the high costs of prolonged treatment will remain a pressing issue for many societies, necessitating layered and resource-adjusted management algorithms as recently suggested, with recognition of the introduced biases as has been detailed (81).

### RCTs versus real world data

Real world data (RWD) should complement clinical trial data for assessing the effectiveness of studied interventions, and RWD confirm the effectiveness of early ARSI use. In general, RWD, better reflecting everyday patients and usual daily practice, show shorter OS and more toxicity than to RCTs.

A recent study of 22 oncology drugs for 29 indications demonstrated a median of 6.3 months shorter survival in the real-world outcomes of Medicare beneficiaries compared to the benefit obtained in the respective pivotal clinical trials. Survival was shorter in 28 of 29 broad cancer treatments, including for abiraterone (before and after docetaxel) and enzalutamide (after docetaxel) (82). In five of seven RCTs comparing an ARSI combination with ADT to ADT (no OS data for ADT only in PEACE-1) the combination treatment arm survival in a large real-life database of Veterans Health Administration (VHA) with *de novo* mCSPC (40.4 months [confidence interval; (CI) 37.7–43.2]) was shorter than the survival of control arm (ADT) trial patients; as detailed above, the latter received suboptimal post-protocol care (Figure 2 and Supplementary Material). The median OS of combination therapy in RCTs greatly exceeded median OS in the veterans’ database (Table 2) (83). In another RWD analysis of patients treated at a university hospital, median OS in high volume mCSPC treated with ADT and either an ARSI (median 42 months) or docetaxel (median 46 months) was also comparable to the survival of control arm patients treated with ADT in the RCTs discussed (Table 2) (84). In a small retrospective analysis from several centers, ADT + ARSI led to a significantly longer rPFS than ADT + docetaxel and ADT alone in patients with newly diagnosed mHSCPC (21, 12, and 13 months, respectively) (85). This was shorter than reported in LATITUDE, with a median rPFS of 33.0 months (20, 21).

RWD also highlights the difference in comorbidity between patients in real-world settings and those entering RCTs, as discussed above. Older patients with more comorbidities are at greater risk of treatment-related complications. In a real-world cohort of 90,087 men with mHSPC treated with ADT + docetaxel + ARSI, cardiovascular risk factors were common as opposed to patients participating in RCTs, with diabetes, heart failure, obesity, and renal impairment reported in 33%, 17%, 25%, and 26%, respectively (86). By contrast, in the STAMPEDE trial, only 4% of patients had a history of myocardial infarction, 1% of cerebrovascular disease, and 4% had a history of heart failure. Thromboembolic events were rare in STAMPEDE, whereas thromboembolic events occurred at a rate of 40–213 per 1,000 person-years in the real-world analysis (18, 19). Ischemic heart disease and arrhythmias were also more frequent in the real-world setting than in ARCHES and STAMPEDE (18, 19, 22, 86).

## Limitations

Several limitations to our review should be recognized. Our analysis is not based on a systematic review, indicating that our findings are more prone to bias and the selective inclusion of studies. Moreover, factors such as informative censoring, to which control arms in open-label studies are especially prone (87), have not been analyzed. Reported data on post-protocol ARSI exposure are often incomplete, necessitating a range of scenario reporting from best- to worst-case (Figure 2 and Supplementary Material), thus allowing only an estimate.

## Conclusion

ARSIs offer important advances for the treatment of men with advanced prostate cancer. Based on the issues with the ARSI RCTs detailed above, the expected magnitude of benefit of early ARSI use is uncertain, with benefit likely overestimated and toxicity underestimated.

For a drug to be considered essential on the World Health Organization Essential Medicines List (WHO-EML), it should at least increase OS by 4–6 months compared to the standard of care (88, 89). In the 2025 WHO-ELM edition, abiraterone (with enzalutamide a therapeutic alternative) is listed as an essential drug for the treatment of *mCRPC* (90). Several recent observational studies (91–93) demonstrate relatively limited early ARSI use in clinical practice even in high income countries, and studies attribute this to differing guideline interpretation and safety/tolerability concerns. We feel that the uncertainty of the magnitude of benefit of early ARSI use allows for individualization and nuance as opposed to strict routine early use in patient care. This should be reflected in policy decision-making and, as stated above and suggested before (81), should allow for layered societal guideline recommendations recognizing the limitations of the underlying evidence as detailed here. Retaining ARSIs for later and shorter use in patients presenting with mCSPC without a contraindication for chemotherapy remains justifiable (94). In this light, conceding the limitations of comparing the outcome of different arms within the STAMPEDE protocol, comparison of the ADT + abiraterone to the ADT + docetaxel arms failed to show a difference in OS, symptomatic skeletal events, or rate of high-grade toxicity in patients with high-risk non-metastatic castration-sensitive prostate cancer (nmCSPC) or mCSPC (95).

RCTs should provide drug regulatory agencies, health technology assessment agencies, and physicians with reliable data on which to make choices based on the principle of value-based healthcare, with “value” in healthcare defined as the outcome of an intervention relative to its costs (96). In this review, we show that, despite the intensive efforts of investigators, pharmaceutical companies, and academic institutions to conduct RCTs for investigating earlier ARSI implementation, uncertainty remains regarding the true magnitude of benefit due to the RCT design issues detailed above. This remains largely unaddressed in contemporary expert opinion reviews regarding early ARSI use (97, 98). The issues detailed here occur increasingly in contemporary cancer clinical trials, and the recognition of this is important both for physicians guiding their patients traversing the increasingly complex cancer management and in treatment guideline development in general (2, 99).
