# Supplementary material for: Uncertain magnitude of benefit of early second-generation androgen receptor antagonist treatment in advanced prostate cancer
Source: Oncol Rev. 2026 May 12;20:1750202. doi: 10.3389/or.2026.1750202 (PMC13201429; doi:10.3389/or.2026.1750202)
Supplement: Supplementary file 1 [file Table1.DOCX]

**SUPPLEMENTARY MATERIAL.** Post-trial treatment

| **Trial** | **Treatment allocation** |  | **(N)** | **Denominator for post-protocol care** | **Any ARSI post-trial** | **Abiraterone post-trial** | **Enzalutamide post-trial** | **Docetaxel**  **post-trial** | **Cabazitaxel**  **Post-trial** | **Median duration of treatment** |
| --- | --- | --- | --- | --- | --- | --- | --- | --- | --- | --- |
| **Metastatic CRPC, progressing after chemotherapy** | | | | | | | | | | |
| COU-AA-301 ^10,12^ | ADT, Abiraterone, prednisone  ADT, placebo,  prednisone |  | 797  398 | NS  NS | NS  NS | NS  NS | NS  NS | NS  NS | NS  NS | 7.4 (range  0.2-25.6) months  3.6 (range  0.1-24.9) months |
| AFFIRM ^13^ | Enzalutamide  Placebo (prednisone permitted, not required) |  | 800  399 | 336 (42%) *  245 (61%) * | NS  NS | 167/336 (50%)  97/245 (40%) | NS  NS | 68/336 (20%)  57/245 (23%) | 78/336 (23%)  55/245 (22%) | 8.3 months  3.0 months |
| **Metastatic CRPC, before chemotherapy** | | | | | | | | | | |
| COU-AA-302 ^14,15^ | Abiraterone, prednisone |  | 546 | 365 (67%) ** | NS | 69/365 (19%) | 87/365 (24%) | 311/365 (85%) | 100/365 (27%) | 13.8 months (IQR 8.3-27.4 |
|  | Placebo,  prednisone ¶ |  | 542 | 435 (80%) ** | NS | 238/435 (55%) | 54/435 (12%) | 331/435 (76%) | 105/435 (24%) | 8.3 months (3.8-16.6) |
| PREVAIL  ^16,17^ | Enzalutamide |  | 872 | 583 (67%) ** | 378/583 (65%) | 362/583 (62%) | 53/583 (9%) | 481/583 (83%) | 151/583 (26%) | 17.7 months |
|  | Placebo |  | 845 | 695 (82%) ** | 574/695 (83%) | 456/695 (66%) | 364/695 (52%) | 546/695 (79%) | 210/695 (30%) | 4.6 months |

CRPC: castration resistant prostate carcinoma, PCa: prostate carcinoma SOC: standard of care group, NS: Not specified.

For determining the denominator for post-protocol care the latest available published data were used per study extracted either from the main manuscript or from the supplemental data. When the number of patients receiving post-protocol therapy was higher than the number reported in the manuscript as ‘disease progression’, the number of patients receiving post-protocol care was used, depicted by *. When the number of patients receiving post-protocol care was lower than the number reported as progressing, the latter number was used and is depicted with **.

¶ At the final analysis of the COU-AA-302 trial, 238 (44%) patients from the placebo group had subsequently received abiraterone acetate plus prednisone. Of these 238 patients, 93 crossed over from receiving prednisone to abiraterone acetate plus prednisone per the protocol amendment, with the remaining 145 patients receiving abiraterone acetate plus prednisone as subsequent therapy, independent of study amendments.

**SUPPLEMENTARY MATERIAL.** Post-trial treatment (Continued)

| **Trial** | **Treatment allocation** | **(N)** | **Denominator for post-protocol care** | **Any ARSI post-trial** | **Abiraterone post-trial** | **Enzalutamide post-trial** | **Docetaxel**  **post-trial** | **Cabazitaxel**  **Post-trial** | **Median duration of treatment** |
| --- | --- | --- | --- | --- | --- | --- | --- | --- | --- |
| **Metastatic Hormone-Sensitive (mCSPC), or locally advanced PCa** | | | | | | | | | |
| STAMPEDE  ^18,19^ ^$^ | Abiraterone, prednisone, ADT | 501 | 282 (56%) ** | NS | 10/282 (3.5%) | 46/282(16%) | 137/282 (49%) | NS | 33.2 months ¶ |
|  | Placebo, ADT | 502 | 437 (87%) ** | NS | 131/437 (30%) | 157/437 (36%) | 199/437 (46%) | NS | NS |
| LATTITUDE ^20,21^ | Abiraterone, Prednisone, ADT | 597 | 254 (43%) ** | NS | 18/254 (7%) | 57/254 (22%) | 144/254 (57%) | 25/254 (10%) | 25.8 months (IQR 12.3-49) |
|  | Placebo, ADT^%%^ | 602 | 388 (65%) ** | NS | 84/388 (22%) | 99/388 (26%) | 212/388 (55%) | 50/388 (13%) | 14.4 months (IQR 7.3-25.8) |
| ARCHES ^22,23^ | Enzalutamide, ADT | 574 | 131 (23%) * | 33/131^&^ (25%) | 26/131 (20%) | 7/131 (5%) | 48/131 (37%) | 11/131 (8%) | 40.2 months |
|  | Placebo, ADT^ | 576 | 221 (38.4) * | 103/221 (47%)^&^ | 42/221 (19%) | 61/221 (28%) | 71/221 (32%) | 6/221 (3%) | 13.8 months |
| ENZAMET ^25-27^ | Enzalutamide first line, ADT | 563 | 268 (61%) * | 268/268 (100%) | 70/268 (26%) | 0/268 (0%) | 47/268 (18%) | 57/268 (21%) | 58 (IQR 49-67) months |
|  | ADT | 562 | 413 (85%) * | 313/413 (76%) | 148/413 (36%) | 205/413 (50%) | 79/413 (19%) | 104/413 (25%) | 23 (IQR 21-25) months |

For determining the denominator for post-protocol care the latest available published data were used per study extracted either from the main manuscript or from the supplemental data. When the number of patients receiving post-protocol therapy was higher than the number reported in the manuscript as ‘disease progression’, the number of patients receiving post-protocol care was used, depicted by *. When the number of patients receiving post-protocol care was lower than the number reported as progressing, the latter number was used and is depicted with **.

^$^In the STAMPEDE trial patients with mCSPC were included in this analysis. ^%%^In LATTITUDE 72 patients in the placebo arm were crossed over to abiraterone before progressing.
^%^ In the PEACE-1 trial, patients were randomized to receive abiraterone with androgen deprivation therapy (ADT), with or without docetaxel and/or radiotherapy. As the supplementary appendix provides data only for the docetaxel-treated (intention-to-treat) group, this is the only dataset included in the table.

| **Trial** | **Treatment allocation** | **(N)** | **Denominator for post-protocol care** | **Any ARSI post-trial** | **Abiraterone post-trial** | **Enzalutamide post-trial** | **Docetaxel**  **post-trial** | **Cabazitaxel**  **Post-trial** | **Median duration of treatment** |
| --- | --- | --- | --- | --- | --- | --- | --- | --- | --- |
| **Metastatic Hormone-Sensitive (mCSPC), or locally advanced PCa (continued)** | | | | | | | | | |
| PEACE 1 ^24^ | Abiraterone, prednisone, ADT, docetaxel | 347 | 141 (41%)* | 65/141  (46%) | 22/141 (16%) | 57/141 (40%) | 29/141 (21%) | 84/141 (60%) | 34.1 months (95% C.I. 30.0-  43.5 months) |
|  | ADT, docetaxel | 350 | 263 (75%) *^%^ | 213/263 (81%) | 153/263 (58%) | 119/263 (45%) | 25/263 (10%) | 114/263 (43%) | NS |
| TITAN^30,93^ | Apalutamide + ADT | 525 | 138 (26%) **^,&^ | 29/138 (21%)^&^ | 20/138 (14%) | 9/138 (7%) | 37/138 (27%) | 2/138 (1%) | 39.3 (range 0-55.7) months |
|  | Placebo + ADT^@@^ | 527 | 261 (50%) ** | 76/261 (29%)^&^ | 56/261 (21%) | 20/138 (8%) | 71/261 (27%) | 4/261 (2%) | 20.2 (range 0.1-37.0) months |
| ARASENS^28^ | Darolutamide + docetaxel + ADT | 651 | 352 (54%)** of * | 112/352 (29%)^&^ | ^@^ | ^@^ | ^@^ 52/352 (15%) | | 41.0 (range 0-57.6) months |
|  | Placebo + docetaxel + ADT | 654 | 526 (80%)** of * | 290/526 (55%)^&^ | ^@^ | ^@^ | ^@^ 71/526 (13%) | | 16.7 months. |
| ARANOTE ^29^ | Darolutamide + ADT | 446 | 128 (54%)** | 32/128 (25%)^&^ | 26/128 (20%) | 6/128 (5%) | 46/128 (36%) | 2/128 (2%) | 24.2 months |
|  | Placebo + ADT | 223 | 94 (80%)** | 33/94 (35%)^&^ | 21/94 (22%) | 12/94 (13%) | 46/94 (49%) | 1/94 (1%) | 17.3 months |

For determining the denominator for post-protocol care the latest available published data were used per study extracted either from the main manuscript or from the supplemental data. When the number of patients receiving post-protocol therapy was higher than the number reported in the manuscript as ‘disease progression’, the number of patients receiving post-protocol care was used, depicted by *. When the number of patients receiving post-protocol care was lower than the number reported as progressing, the latter number was used and is depicted with **.

^&^In the ARCHES, TITAN, and ARASENS trial the number of *first* life prolonging treatment were detailed allowing to determine the exact percentage of patients receiving an ARSI post progression in first-line.
^@^In ARASENS the abiraterone and enzalutamide were not reported separately, and docetaxel and cabazitaxel were not reported separately. ^In ARCHES 184 patients in the placebo arm were crossed over to enzalutamide before progressing.
 ^@@^In TITAN 208 patients in the placebo arm were crossed over to apalutamide before progressing.

**SUPPLEMENTARY MATERIAL.** Post-trial treatment (Continued)

| **Trial** | **Treatment allocation** | **(N)** | **Denominator for post-protocol care** | **Any ARSI post-trial** | **Abiraterone post-trial** | **Enzalutamide post-trial** | **Docetaxel**  **post-trial** | **Cabazitaxel**  **Post-trial** | **Duration of treatment** |
| --- | --- | --- | --- | --- | --- | --- | --- | --- | --- |
| **Prostate cancer with biochemical recurrence or nmCRPC** | | | | | | | | | |
| PROSPER ^31,32^ | Enzalutamide, ADT | 930 | 310 (33%)* | NS | 152/310 (49%) | 44/310 (14%) | 185/310 (60%) | 46/310 (15%) | 33.9 months |
|  | Placebo, ADT | 465 | 303 (65%)* | NS | 178/303 (59%) | 109/303 (36%) | 141/303 (47%) | 49/303 (16%) | 14.2 months |
| EMBARK ^35^ | Enzalutamide + leuprolide  Leuprolide + placebo  Enzalutamide monotherapy | 355  358  355 | 58 **  140 **  84 ** | NS  NS  NS | NS  NS  NS | NS  NS  NS | NS  NS  NS | NS  NS  NS | 38.7 months (range 0.1 to 88.9) in all groups |
| SPARTAN ^33^ | Apalutamide + ADT | 806 | 386* | 314/386 (81%) | 282/386 (73%) | 32/386 (8%) | Ubknown how many eventually received | Ubknown how many eventually received | 32.9 months (range 0.1-74.5) |
|  | Placebo + ADT | 401 | 285* | 244/285 (86%) | 206/285 (72%) | 38/285 (13%) | Ubknown how many eventually received | Ubknown how many eventually received | 11.5 months (range 0.1-37.2) |
| ARAMIS ^58^ | Darolutamide + ADT | 955 | 141* | NS | 29/141 (21%) | 28/141 (20%) | 81/141 (57) | 1/141 (0%) | 25.8 months |
|  | Placebo + ADT | 554 | 137* | NS | 33/137 (24%) | 29/137 (21%) | 75/137 (55%) | 0 | 11.6 months |

For determining the denominator for post-protocol care the latest available published data were used per study extracted either from the main manuscript or from the supplemental data. When the number of patients receiving post-protocol therapy was higher than the number reported in the manuscript as ‘disease progression’, the number of patients receiving post-protocol care was used, depicted by *. When the number of patients receiving post-protocol care was lower than the number reported as progressing, the latter number was used and is depicted with **.
